# Work-related musculoskeletal disorders among gig-based food delivery workers: a systematic review and meta-analysis

**DOI:** 10.3389/fpubh.2026.1788523

**Published:** 2026-03-23

**Authors:** Yousef Alrashidi, Shyamkumar Sriram, Mohammed A. Beek, Hammad A. Fadlalmola, Muayad Albadrani

**Affiliations:** 1 Department of General and specialized surgery, College of Medicine, Taibah University, Madinah, Saudi Arabia; 2 Department of Rehabilitation and Health Services, College of Health and Public Service, University of North Texas, Denton, TX, United States; 3 College of Medicine, Taibah University, Madinah, Saudi Arabia; 4 Department of Community Health Nursing, Nursing College, Taibah University, Madinah, Saudi Arabia; 5 Department of Family and Community Medicine and Medical Education, College of Medicine, Taibah University, Madinah, Saudi Arabia; 6 Health and Life Research Center, Taibah University, Madinah, Saudi Arabia

**Keywords:** food delivery workers, gig economy, meta-analysis, occupational hazards, occupational medicine, prevalence, systematic review, work-related musculoskeletal disorders

## Abstract

**Background:**

The digital economy has spurred gig work, especially in food delivery, which grew during COVID-19. However, gig workers face occupational hazards like traffic accidents, poor ergonomics, and unsafe conditions, leading to work-related musculoskeletal disorders (WMSDs). Studies show high rates of back and neck pain among delivery riders due to physical strain, repetitive motions, and long hours. WMSDs reduce productivity and increase healthcare costs. This review examines WMSD prevalence, risk factors, and related issues like accidents and violence among food delivery workers.

**Methods:**

We searched for relevant articles up to June 2025 from PubMed, Scopus, and Web of Science. Two independent reviewers extracted data from the selected studies, including baseline information, outcomes, and prevalence of WMSDs. All data analyses were performed using R version 4.3.3.

**Results:**

After removing 1,013 duplicate records, we retained 1,279 for screening. Following a thorough review, we identified 23 eligible entries for inclusion in our study. As per our analysis, delivery workers face high injury prevalence: lower back (43%), shoulder (39%), neck (30%), upper back (24%), and RTA (25%). Risk factors include gig economy systemic vulnerabilities, prolonged static postures, vibration exposure, open-door vehicles, and dangerous traffic practices. Different forms of violence (physical, verbal, and psychological) affected delivery workers, while exploitation and discrimination were particularly evident among minorities.

**Conclusion:**

This review demonstrated a high burden of WMSDs among delivery workers, who face serious hazards like injuries, accidents, and violence due to precarious gig economy conditions, time pressures, and poor safety measures. This study provides the first quantitative pooled estimates of WMSD prevalence among food delivery workers, along with an additional narrative synthesis of traffic accidents and workplace violence.

## Introduction

The gig economy has expanded rapidly over the past decade, with an estimated 435 million workers worldwide engaged in some form of platform-based or freelance labor. In the United States alone, approximately 36% of the workforce participates in gig work, while similar trends have been reported across Europe, China, India, and Southeast Asia. This growing workforce is increasingly exposed to occupational health risks, particularly musculoskeletal disorders (MSDs), which represent one of the leading causes of disability and work-related morbidity globally (1). Previous studies have reported high prevalence of MSDs among gig workers, with estimates ranging from 30% to over 60%, depending on job type, working conditions, and exposure to physical and psychosocial stressors (2, 3).

The digital economy has transformed global labor markets through the emergence of gig businesses, signaling a significant global shift in market dynamics where jobs are performed through electronic platforms (4). Digital platforms not only offer job opportunities for gig workers but also imply a structural shift in labor organization within the food service industry that has gained a rapid and significant growth, as it satisfies immediate cravings without requiring cooking (5, 6). Food delivery services proved to be vital to avert crowded places during the COVID-19 pandemic, and many consumers began to rely on services provided by online digital platforms, which led to a growing food delivery market (7).

Multiple occupational safety and health risks affect workers in the gig industry. Food delivery workers encounter multiple physical hazards, leading to different injuries such as falls, slip-and-trip incidents, and road-related accidents from traffic collisions (8, 9). The prevalence of occupational hazards among delivery riders has significantly increased due to poor ergonomic practices, such as improper posture and lifting techniques during deliveries, long hours spent on the road, and chronic fatigue (10). Working in unpredictable and hazardous environments: adverse weather conditions or poorly maintained roadways add to those risks (11). Exacerbating the vulnerability is the fact that many delivery workers do not have access to adequate healthcare services (12). Furthermore, the requirement to work under unsafe conditions, lack of job security, and proper training have a large influence on the prevalence of occupational hazards (13).

The prevalence of work-related musculoskeletal disorders (WMSDs) among food delivery workers is growing. A Malaysian study demonstrated that 73% of food delivery workers suffer from lower back pain, possibly attributed to the physical demands of lifting and transporting food items (10). Moreover, neck pain linked to awkward postures was reported in 53% of workers while riding, and 48.7% experienced upper back pain due to strain from prolonged riding positions and carrying heavy delivery bags, as well as the repetitive motions involved in handling food packages.

The presence of WMSDs negatively impacts both the quality of life and job performance, through decreased productivity and increased absenteeism (14). Prevalence of WMSDs indirectly may contribute to economic burdens due to the treatment and management of chronic pain, burdening both individuals and the healthcare system (10).

From a conceptual perspective, psychosocial working conditions in the gig economy may play a central causal role in shaping health outcomes among food delivery workers. Platform-based employment is typically characterized by job insecurity, income dependence, algorithmic management, and strict performance metrics, which collectively expose workers to chronic psychological stress and intense time pressure. Recent conceptual and empirical studies have emphasized that the design of gig work itself may generate latent health risks through sustained exposure to psychosocial stressors and emerging risk factors inherent to digital labor platforms (15, 16). These psychosocial stressors often translate into prolonged working hours, insufficient rest, sleep deprivation, and mental fatigue. In turn, fatigue and sustained stress can impair cognitive functioning, attention, and risk perception, increase biomechanical load through prolonged static postures and repetitive movements, and promote risky behaviors such as speeding or neglecting safety measures. This pathway suggests a plausible causal chain whereby adverse psychosocial working conditions contribute to fatigue, which subsequently increases vulnerability to musculoskeletal disorders, traffic accidents, and broader physical and psychological health outcomes.

Building on this framework, Taylor et al. systematically suggests that gig work exposes workers to a combination of physical hazards (such as ergonomic strain and accident risk) and psychological hazards (including algorithmic control, economic insecurity, and performance surveillance), which together contribute to work intensification, effort–reward imbalance, and normalization of occupational risk (17). These mechanisms may lead workers to accept unsafe working conditions, extend working hours, compromise rest and recovery, and internalize risk as an inherent feature of platform-based labor. This conceptual model provides a useful lens to understand how the structural design of food delivery work may causally shape both musculoskeletal and broader health outcomes.

A previous review addressed only poor imbalance, regulatory failures, and platform practices in the gig economy (17). While previous reviews have examined occupational hazards and health risks in the gig economy more broadly, no prior study has quantitatively synthesized pooled prevalence estimates of work-related musculoskeletal disorders specifically among food delivery workers. Therefore, this systematic review and meta-analysis aims to provide a quantitative synthesis of WMSD prevalence, complemented by a narrative synthesis of road traffic accidents and workplace violence in this occupational group.

## Methods

Following the Cochrane Handbook for Systematic Reviews of Interventions and the PRISMA guidelines, we conducted this meta-analysis to examine the prevalence and risk factors of work-related musculoskeletal disorders (WMSDs), accidents, and violence among food delivery workers (18, 19).

### Eligibility criteria

The inclusion and exclusion criteria were predefined prior to conducting the literature search and were applied consistently during the screening and eligibility assessment stages.

Inclusion criteria:

- Cross-sectional studies assessing the prevalence of WMSDs among delivery workers.
- Relevant articles reported in English.
- Articles that could be downloaded in full text.

Exclusion criteria:

- Articles that do not explicitly provide prevalence estimates.
- All letters to editors, opinions, and comments.
- Abstracts and reviews.

### Information sources and search strategy

A multi-step process was used to retrieve records and gather relevant evidence. It began with a broad search using generic terms across PubMed, Scopus, and Embase to identify initial articles during the pilot phase to refine search terms. The final systematic search was conducted across PubMed, Scopus, and Web of Science. Each term was carefully evaluated for relevance, and any redundant ones were discarded. The refined search, incorporating MeSH terms and related keywords, was then systematically applied to PubMed, Scopus, and Web of Science. The final search strategy was as follows: ((Food OR gig) AND (worker OR workers OR “deliverer” OR delivery) OR “takeaway riders”) AND (“musculoskeletal disorders” OR “MSDs” OR “WRMSDs” OR “WMSDs” OR “pain” OR “injuries” OR “discomfort”). Our search was completed in June 2025 with a detailed, specific query. Additionally, we performed forward and backward searches based on the records obtained from the initial search to identify relevant studies further (Supplementary Appendix 1).

### Selection process

The senior author anonymized the records obtained through the specified search method by removing author names and any identifying information to reduce bias. Then, two reviewers independently screened the titles and abstracts of these anonymized records using a predefined checklist. When conflicts occurred, the senior author stepped in. After the initial screening, the same two blinded reviewers evaluated the full texts of the selected articles for eligibility, discussing and resolving any discrepancies.

### Data collection

Full-text articles meeting predetermined criteria were reviewed, and relevant data were extracted into a shared Google spreadsheet. Initially, baseline information was collected, including the author’s last name, study design, country, and outcomes. Additionally, details such as sample size, age, gender distribution, BMI, working hours, job experience, and vehicle types were gathered. For analysis, we extracted data on the prevalence of various musculoskeletal disorders, such as low back, shoulder, upper back, and neck injuries.

### Outcome measures

#### Prevalence of occupational injuries

Prevalence estimates were extracted as reported in the original studies, most commonly referring to symptoms occurring within the 12 months preceding the questionnaire. However, recall periods and measurement instruments varied across studies, including self-reported surveys, the Nordic Musculoskeletal Questionnaire, and study-specific tools.

#### Risk factors for accidents

Different risk factors that explain the prevalence of traffic accidents among delivery workers were carefully looked for and listed.

#### Risk factors for musculoskeletal disorders

To provide context, we synthesized potential risk factors for WMSDs among delivery workers whenever clearly provided in the included texts.

#### Forms of violence against delivery workers

We documented various forms of violence perpetrated against delivery workers, categorizing them into physical, verbal, or psychological.

### Risk of bias

A thorough quality assessment of the cross-sectional studies was carried out using the NIH Quality Assessment Tool for Observational and Cohort Studies (20). Before the formal review, two independent reviewers piloted the tool on five randomly chosen studies, discussed any discrepancies, and refined their interpretation criteria to ensure consistent scoring. All following assessments were done independently by both reviewers, with disagreements resolved by a third reviewer. It is important to note that although the NIH tool offers a solid framework for bias assessment, 4 of its 14 domains are not applicable to cross-sectional studies. Therefore, studies rated as “Fair” under this tool would probably be rated as “Good” if these specific domains were excluded from the scoring.

### Statistical analysis

The study analyzed the prevalence of work-related musculoskeletal disorders (WMSDs) among delivery workers using R v 4.3.3. We used the metaprop function from the meta package to pool estimates for lower back, shoulder, neck, upper back, and road traffic accident (RTA) prevalence rates. Results were presented as pooled proportions with 95% confidence intervals (CIs), and statistical significance was defined as a *p*-value below 0.05. To evaluate heterogeneity across studies, the *I*^2^ statistic was applied, with values exceeding 40% and a *χ*^2^ *p*-value under 0.1 considered indicative of significant variation. Due to differences in study methodologies and observed heterogeneity, a random effects model was employed. The restricted maximum likelihood (REML) method estimated heterogeneity (*τ*^2^), and findings were visualized using a forest plot, which displayed individual study data alongside overall effect measure variability.

## Results

### Study selection

**Figure 1:**
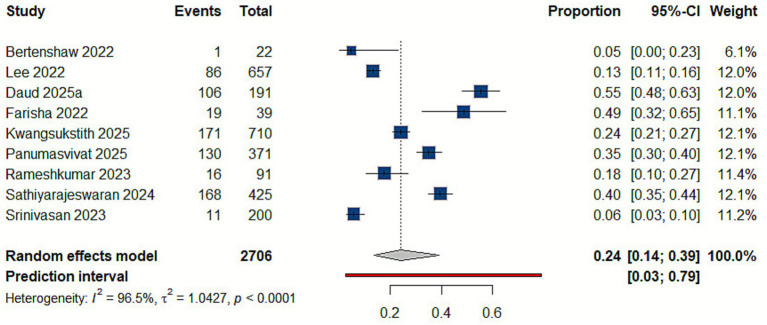
PRISMA flow diagram of the study selection process.

After searching four databases, we collected 2,292 records. Removing 1,013 duplicates left us with 1,279 unique records. We then screened the titles and abstracts, excluding 1,229 entries. We retrieved the full text of the remaining 50 records and evaluated them against our eligibility criteria. During this evaluation, we excluded eight non-English articles, two review articles, three editorial comments, three studies not providing prevalence estimates, and 11 that were not targeting food delivery workers. Eventually, 23 articles were included in the review. The flow diagram for study selection is shown in Figure 1.

### Characteristics of the included studies

This review consisted of 23 cross-sectional studies (9, 10, 21–41), seven from Thailand, three from India, two from Malaysia, two from China, and one each from Korea, Australia, Italy, Canada, Brazil, the United States, and the United Kingdom. The total number of subjects was 150,635. Their age averaged 43.39 (±7.47) years, and 67.95% were males. From studies where education data of the participants was properly stated, 15.07% of the participants had an education level below secondary school, while 38.66% had above secondary education. Most of the workers rode motorcycles (88.1%), moped or e-bikes (0.45%), the remainder used cars/vans (0.66%), or bicycles (0.16%). From 14 studies that provided sufficient data, the weighted average of working time for food delivery workers was 7.9 h a day (9, 10, 21, 23, 27–30, 32–34, 36, 38, 40), with the highest working times reported from Malaysia (10.2 h), Thailand (9.8 h), and Brazil (8.5 h). Table 1 completes the depiction of baseline characteristics.

**Table 1:** Summary and baseline characteristics of the included.

Study ID	Design	Country	*N*	Age		Male		BMI	Job duration	Type of vehicle	Educational level	Experience	Conclusion
				mean	SD	n	%		hours				
Bertenshaw (22)	Cross-sectional	Australia	22	24.5	6.5	18	81.8	NR	NR	Bicycle (electric): 5 Bicycle (unpowered): 8 Motorcycle: 4 Scooter (electric): 5	NR	NR	“The majority of FDRs presenting with injuries are not Australian citizens and less than half were Medicare eligible potentially contributing to inadequate access to care especially fracture follow-up. There were spikes in injuries occurring at night, weekends and during periods of pandemic associated lockdowns demonstrating an increased usage of delivery services during these times. Results highlight injury patterns experienced by delivery riders and potentially modifiable risk factors for this rapidly growing area of employment within the gig economy.”
Boniardi (9)	Cross-sectional	Italy	240	30	7	233	97	NR	Less than 3 h: 10 3 to 4 h: 47 5 to 6 h: 60 7 to 8 h: 61 More than 8 h: 56	E-bike: 111 Traditional bicycle: 96 Moped/motorcycle: 21 Other: 6	Degree/master’s degree or higher: 28 High school: 97 Middle school or primary school: 102 Other: 3	<12 months: 47 12 to 36 months: 151 >36 months: 37	“The findings underscore food delivery riders’ complex challenges, emphasizing the need for targeted interventions. The study calls for collaborative efforts between policymakers, employers, OSH professionals, and stakeholders to enhance OSH standards and promote decent working conditions, aligning with the 2030 Agenda for Sustainable Development.”
Champahom (23)	Cross-sectional	Thailand	2000	36.9	8.8	1724	86.2	NR	1–4 h: 64 5–8 h: 488 9–12 h: 1134 More than 12 h: 316	NR	NR	<1 year: 2 participants 1–2 years: 18 participants 3–5 years: 262 participants >5 years: 1720 participants	“The study revealed several key factors influencing crash injury severity among delivery riders. Rider age, particularly the 35–44 age group, showed varying effects on injury severity across the population. Riding experience, contrary to some previous findings, was associated with an increased likelihood of severe injuries, possibly due to over confidence or exposure to more challenging conditions.”
Christie (24)	Cross-sectional	United Kingdom	339	29.7	6.9	249	78	NR	Gig workers: ≈ 5.3 h/day Employed workers: ≈ 2.6 h/day	125 cc: 208 50 cc: 70 126–400 cc: 29 Over 400 cc: 10	NR	NR	“The aim of this study was to compare road injury risks experienced by people who deliver food on motorcycles in relation to the business model in which they work and identify solutions for managing their safety. Our research shows that there are significant differences between the experience of risks and working conditions for people who access work via digital platforms compared to those who are directly employed by restaurants.”
Daud (10)	Cross-sectional	Malaysia	191	27.6	5.7	177	92.7	NR	10.2 ± 2.33 h	Sedan motorcycle: 160 Scooter: 30 High-powered motorcycle: 1	NR	1–6–12 months: 92 2- > 12 months: 99	“The study highlighted a medium risk of WMSDs among food delivery riders. Ergonomic interventions, particularly in motorcycle design, are necessary to mitigate these risks and improve occupational safety and health.”
Daud (25)	Cross-sectional	Malaysia	191	27.6	5.7	177	92.7	26.01 ± 6.54 kg/m^2^	10.2 ± 2.33 h	Sedan motorcycle: 160 Scooter: 30 High-powered motorcycle: 2	Non-formal: 5 Primary school: 4 Secondary school: 84 Form 6/STAM/diploma: 76 Degree/master’s/PhD: 22	6–12 months: 92 >12 months: 100	“Targeted interventions are essential to mitigate ergonomic risks and enhance rider safety. Occupational health policies should prioritise pre-work exercise and WBV exposure reduction to minimise musculoskeletal strain. Future longitudinal studies are needed to evaluate the long-term impact of these risks on riders’ health.”
Daud et al. (51)	Cross-sectional	Malaysia	39	28	6.96	38	97.4	Underweight (<18.5): 1 Normal (18.5–24.9): 18 Overweight (25–29.9): 7 Obese (>30): 13	Less than 9 h: 24 9 to 15 h: 15	NR	Bachelor’s degree: 16 Secondary education or lower: NR	6 to 12 months: 16 13 to 24 months: 16 25 to 36 months: 7	“This study was conducted with the intention to assess the prevalence of back pain among food delivery riders. The highest 12 months’ prevalence was low back pain (59.0%), while for 7 days’ prevalence was led by lower back pain and shoulder pain (59.0%, respectively). This was followed by shoulder and neck pain with 53.8%, respectively and upper back pain with 48.7%.”
Jing (26)	Cross-sectional	China	5,746	34	7	5,537	97	NR	NR	NR	Education level (years): mean = 11.098, SD = 1.931. Specific categories (e.g., high school, college) not enumerated.	NR	“Although many food-delivery riders find benefits in the flexibility of this work, the work conditions hide many risks without protection, such as health insurance. Based on this realistic background, this study tests whether income dependence contributes to work injuries among delivery riders by adopting a mixed-method approach and powerdependence theory. Specifically, we show that income dependence has a positive predictive effect on work injuries, and workload plays a mediating role. In addition, we found a significant difference in the effect of income dependence on the workload of riders with different levels of difficulty in obtaining subsidies, such that the more difficult it is for riders to obtain subsidies, the more obvious is the positive effect of income dependence on workload. We believe that these findings will inform future research and practices to improve the well-being of delivery riders.”
Kwangsukstith (28)	Cross-sectional	Thailand	709	32.9	8.6	487	68.7	25.63 ± 5.29 kg/m^2^	≥8 h/day: 547 <8 h/day: 162	Motorcycle: 709	Primary school: 35 Secondary school: 325 Undergraduate: 110 Graduate and higher: 239	>1 year: 483 ≤1 year: 226	“Countries such as Vietnam and China, which reported higher rates before the COVID-19 pandemic. Notably, Malaysia reported higher rates conducted during the COVID-19 pandemic. Factors contributing to these accidents include red-light running, wrong-way riding, concerns about customer behaviors, and inadequate sleep. The pressure to meet delivery deadlines customer expectations for timely service, and irregular work schedules exacerbate these risks, leading to risky riding behaviors. Additionally, inadequate sleep and fatigue impair cognitive function, reaction times, and impaired judgment on roads. The high-risk environment of the gig economy heightens the likelihood of accidents.”
Kwangsukstith (27)	Cross-sectional	Thailand	710	32.6	8.5	487	68.7	Underweight (<18.5): 33 Normal (18.5–22.9): 221 Overweight (23–24.9): 119 Obese (≥25): 336	Median 7.7 h per day	Motorcycle: 710	Primary school or less: 35 Secondary school: 325 Diploma: 110 Bachelor’s degree or higher: 239	Median 24 months	“The high prevalence of occupational hazards and health issues among MFDRs is worrisome. It is critical for platform companies and health sectors to introduce effective protective measures for workers, including establishing health surveillance, and supplying PPE.”
Laskaris (29)	Cross-sectional	United States	1,650	34.4	9.1	1,279	77.5	NR	<20 h per week: 617 20–39 h per week: 568 ≥40 h per week: 465 participants	Car: 969 Moped or e-bike: 681	NR	< 1 year: 314 participants 1 to 2 years: 411 participants 2 to 3 years: 381 participants 3 to 4 years: 168 participants ≥4 years: 376 participants	“In NYC and other cities, food delivery gig work is more likely to be a worker’s main source of income than a “flexible” side hustle, despite platform company narratives. The results of this and existing studies suggest that the deliver-at-all-cost reality that is needed to survive financially as a fully dependent food delivery gig worker creates a perfect storm for occupational injury and, to a lesser extent, assault.”
Li et al. (30)	Cross-sectional	China	657	27.1	6.75	466	70.90%	Underweight (<18.5): 65 Normal (18.5–23.9): 373 Overweight (24–27.9): 153 Obese (≥28): 66	< 8 h: 302 8–10 h: 263 11–13 h: 69 ≥14 h: 23	Bicycles: 72 Motorcycles/battery-powered bikes: 539 Vans/cars: 37 Other: 9	Junior high school or below: 140 Senior high school: 154 Junior college: 159 Bachelor’s degree or above: 204	<5 years: 493 ≥5 years: 164	“MSDs were common among takeaway riders. Personal lifestyles (meal irregularity) were found to predict the occurrence, while work-related Frontiers in factors (longer years of employment and prolonged food delivery distance) were positively associated with severity. Should be made to prevent MSDs in this population.”
Molo (32)	Cross-sectional	Thailand	257	34	-	220	85.60%	NR	≤8 h: 103 >8 h: 154	Modified motorcycles: 20 Standard motorcycles: 237	Primary school: 7 Secondary school: 104 Higher education: 146	≤12 months: 93 13-35 months: 74 ≥36 months: 90	“Based on this model, we recommend several measures to minimize accidents among FDRs: ensuring adequate sleep, implementing work-rest schedules to mitigate fatigue, managing job-related stress effectively, inspecting motorcycle conditions before use, and exercising increased caution when delivering food during rainy conditions.”
Panumasvivat (33)	Cross-sectional	Thailand	454	55.2	9.6	271	60.5	Underweight (BMI < 18.5): 34 Normal weight (BMI 18.5–22.9): 134 Overweight (BMI 23.0–24.9): 90 Obese (BMI ≥ 25.0): 187	Mean 55.2 ± 18.2 h per week	Engine capacity ≤110 cc: 174 Engine capacity >110 cc: 214 Clutch gear: 156 Auto gear: 232	Primary or less: 46 Secondary: 281 Tertiary or more: 120	≤1 year: 84 >1 year: 362	“This study reveals a high prevalence of work-related musculoskeletal disorders among food delivery riders, particularly in the back, neck, and shoulders. Contributing factors include prolonged working hours, awkward postures, repetitive movements, and vehicle-related elements such as engine capacity and delivery bags. These findings highlight the strain on postural muscles caused by prolonged riding. Preventive strategies, including ergonomic motorcycle designs, intervention, and rider posture training, are recommended.”
Prakobkarn (34)	Cross-sectional	Thailand	809	33	9.8	713	88.1	NR	≤8 h: 211 >8 h: 598	NR	Primary/Elementary school: 233 Junior high school: 364 Senior high school: 108 Bachelor’s degree and above: 104	≤6 months: 52 >6 months–1 year: 115 1–3 years: 418 >3 years: 224	“In this study, a survey was conducted on motorcycle accidents among FDDs in urban areas in Bangkok, Thailand. (I) It aimed to explore the prevalence of accidents among FDDs was observed to be 35.1%. (II) The FDDs who participated in this study evinced overall high KAP scores. (III) Our study identified that several influencing factors related to KAP, including driving more than 16 rounds per day on average and following improper practices, were related to motorcycle accidents among FDDs. (IV) Thus, this study, which focuses solely on FDDs from an urban community, showed a correlation between the KAP scores of FDDs with a knowledge score positive significantly with practice score. “
Pratt (35)	Cross-sectional	Canada	6,046	15.8	1.5	3,861	63.9	NR	NR	NR	NR	NR	“Our findings provide occupation group-specific information on common injury types that can be used to support targeted approaches to reduce incidence of youth injury in the workplace.”
Srinivasan et al. (38)	Cross-sectional	India	91	29.1	5.8	NR	NR	NR	NR	NR	NR	NR	“High prevalence of WMSDs among food delivery workers was revealed in this study. However, the results clearly show that participants who work are more likely to experience lower back and shoulder musculoskeletal disorders than other regions of the body.”
Sathiyarajeswaran (31)	Cross-sectional	India	425	269	6.9	425	100	NR	NR	motorcycles: 425	Secondary or below: 58 Higher secondary or diploma: 129 Graduate or post-graduate: 238	<1 year: 128 1 to 2 years: 129 2–5 years: 168 >5 years: 21	“Research in the area of assessing the pain and injuries among food delivery riders is minimal. The present study, notably, extends the understanding of the health impact of full-time food delivery work by revealing a high prevalence of physical pain, particularly in the lower back.”
Siqueira (36)	Cross-sectional	Brazil	563	32.4	8.8	542	96.3	NR	<8 h: 148 From 8 to 10 h: 134 >10 h: 226	Bicycle: 65 Motorcycle: 495	Until complete high school: 172 Complete high school or more: 390	<3 years: 303 4 to 7 years: 120 >7 years: 140	“A total of 563 delivery workers took part in the study. They were predominantly men (96.3%), young, with half of them aged under 33 and almost 80% under 40. Approximately 70% of the delivery workers were mixed-race (47.3%) or black (23%), and had completed high school. There were participants from all regions of Brazil, covering 21 states and the Federal District, with the Northeast (46.2%) and Southeast (36.5%) regions predominating Most participants stated wearing helmet (88%) while working, but 67.6% admit to running red lights in traffic.”
Siu (37)	Cross-sectional	Taiwan	128,234	NR	NR	84,440	65.8	NR	NR	Mopeds (≤50 cc): 3840 Scooters (50 < cc ≤ 250): 121659 Heavy motorcycles (250 < cc < 550): 1925 Heavy motorcycles (≥550 cc): 810	NR	NR	“In conclusion, this study demonstrates that the COVID-19 restrictions was associated with higher odds of injuries and fatalities in motorcycle crashes. The COVID-19 restrictions was associated with an increased proportion of motorcycles involved in traffic crashes, which may indicate the widespread preference for private transportation over public transportation. Furthermore, crashes involving motorcycles used for food delivery have increased, likely due to the growing popularity of point-to-point food delivery services in response to COVID-19 restrictions. These observations underscore the necessity for implementing measures to mitigate the risk of motorcycle crashes during pandemics.”
Srinivasan (38)	Cross-sectional	India	200	26.5	7.34	200	100	NR	≤8 h: 85 >8 h: 115	NR	NR	≤12 months: 71, 35.5% 13-24 months: 114 ≥25 months: 15	“We can conclude that MSDs are common in all three groups, namely pain in the past year, pain in the past week, and activity prevention. This incidence demonstrates that MSDs can impact delivery personnel even at younger ages, which can be avoided by reducing workload and observing adequate safety procedures while working.”
Wanwaen (40)	Cross-sectional	Thailand	253	30.5	7.96	203	80.2	NR	≤8 h: 101 >8 h: 152	NR	Primary/secondary school: 126 Undergraduate: 57 Graduate or higher: 70	1–5 years: 41 6–10 years: 105 11–15 years: 41 >15 years: 66	“The findings could be useful for relevant agencies and organizations in considering safety at work, such as having appropriate working hours and stress prevention to reduce the risk of motorcycle accidents among food delivery riders.”
Yoo (41)	Cross-sectional	Korea	1,000	48.2	10	794	79.4	NR	Designated drivers: 6.08 ± 1.8 h Food-delivery drivers: 7.38 ± 1.9 h Housekeeping managers: 6.69 ± 1.9 h	NR	High school or lower: 664 Two-year college: 267 University or higher: 69	Designated drivers: 3.97 years Food-delivery drivers: 3.08 years Housekeeping managers: 4.96 years	“The results revealed unfavorable working environment and inferior occupational health of platform workers compared with those of the general population.”

NR, non-reported; BMI, body mass index kg/m^2^; FDRs, food delivery riders; OSH, occupational safety and health; MSDs, musculoskeletal disorders; MFDRs, motorcycle food delivery riders.

### Risk of bias

Cross-sectional studies were assessed with the NIH Quality Assessment Tool for Observational Studies, emphasizing design-specific aspects like population representativeness, exposure and outcome measurement, control of confounding variables, and statistical methods. They were rated as “Good,” “Fair,” or “Poor,” reflecting low, moderate, or high bias risk, respectively. All included studies were categorized as fair quality (Supplementary Appendix 1).

### Results of syntheses

Risk factors for road traffic accidents (RTAs) and work-related musculoskeletal disorders (WMSDs) reported in the included 23 studies are summarized in Tables 2 and 3, respectively. Unless otherwise stated, all risk factors and violence-related findings presented in the Results section are derived directly from these included primary studies. Most included studies reported risk factors descriptively without providing extractable effect estimates (e.g., odds ratios with confidence intervals), or reported them using heterogeneous definitions and covariate adjustments that limited comparability. Therefore, we summarize these associations qualitatively in Tables 2 and 3 and interpret them cautiously as correlational findings from cross-sectional evidence.

**Table 2:** Risk factors for road traffic accidents (RTAs) among delivery workers reported in the included studies.

Category	Risk factors
Work-related factors	Choice of transport vehicles, such as motorcycles, motorized scooters, traditional bikes, and electric bikes, and non-enclosed vehicles (9, 22). The risky employment status of workers who are part of the gig economy model of employment or as independent contractors leads to limited worker protections, less tracking of worker accidents, overworking, and a lack of healthcare insurance (9, 22, 24, 29). Complete reliance on platform work increases the vulnerability of workers to injuries, assault, and exploitation (9, 21, 26). Prolonged riding time leading to musculoskeletal strain (33, 34, 40). Employers incentivizing their workers to pursue dangerous traffic practices, such as speeding, running red lights (21), wearing damaged protective equipment (19), riding on icy or wet roads, and carrying unstable packages (24).
Worker-related factors	Poor sleep (28, 32). Young age (36). Male gender (22, 23, 34, 40). Low education and income (23). Immigrants, minorities, and dark-skinned ethnicities (22, 29, 36).

**Table 3:** Risk factors for work-related musculoskeletal disorders (WMSDs) among delivery workers reported in the included studies.

Category	Risk factors
Work-related factors	Poor static posture and prolonged painful positions (neck extension, leaning forward, hands above the shoulders, etc.), carrying heavy backpacks, or due to the type of vehicle (9, 21, 24, 25, 30, 41). Moving heavy or unstable packages and using poor handling techniques (24, 38, 39, 41). Distant shipping locations leading to prolonged rides in static positions (9, 21, 30). Exposure to whole-body vibration (WBV) above the exposure-action value (EAV) limit (10).
Worker-related factors	Irregular meals and dehydration (30, 31). Long working experience (>5 years) (30, 31). Haste to deliver on time (9, 38). Older age (31, 33, 35). Substance use (31).

#### Prevalence of WMSDs

##### Lower back

**Figure 2:**
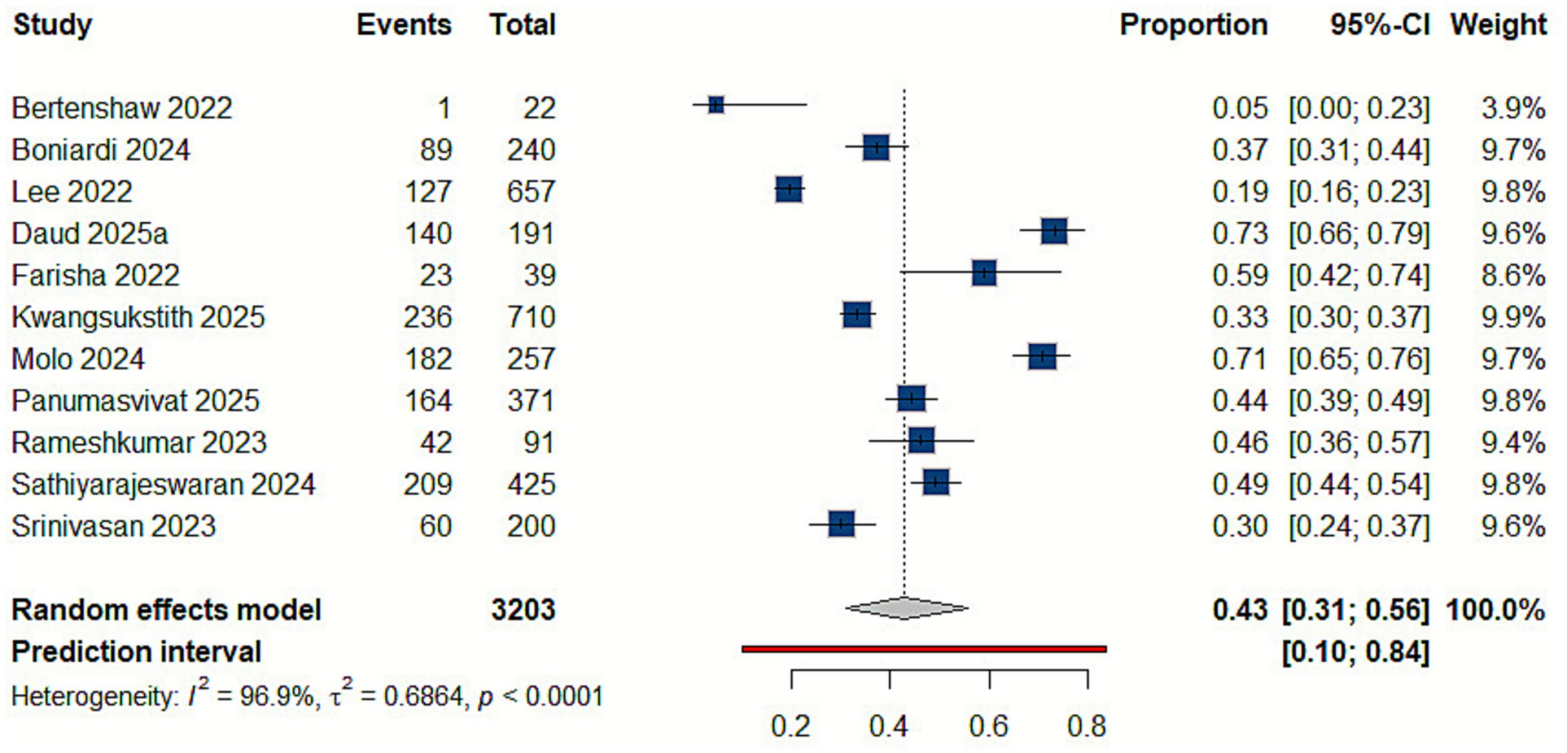
Forest plot of the prevalence of lower back disorders.

The pooled estimates from 11 studies indicate that the prevalence of lower back disorders among delivery workers is 43% (95% CI: 0.31–0.56), reflecting substantial variability across countries, study populations, and working conditions (9, 10, 21, 22, 27, 30–33, 38, 39) Figure 2.

##### Shoulder

**Figure 3:**
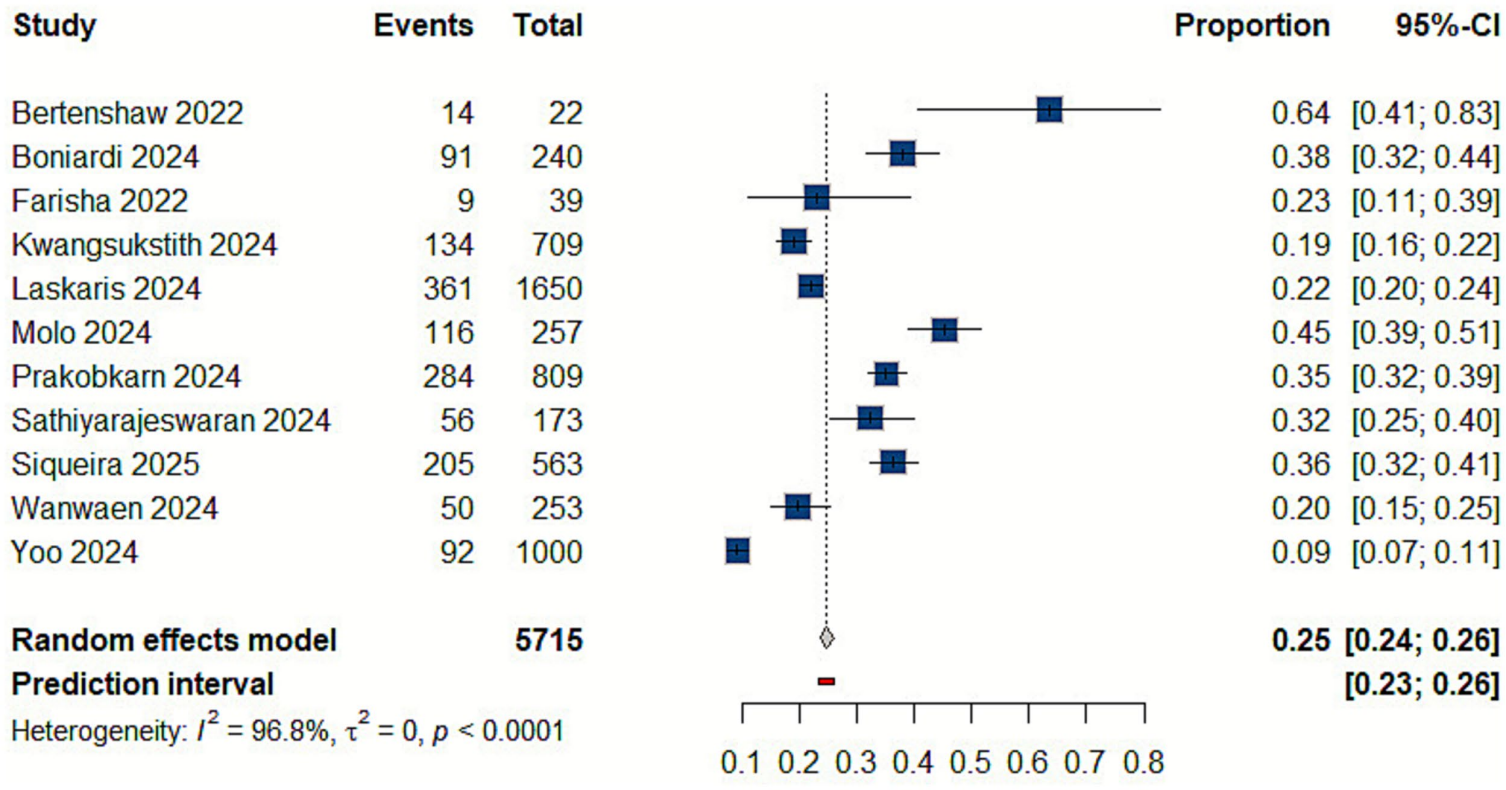
Forest plot of the prevalence of shoulder disorders.

Eleven studies reported shoulder disorders (10, 21, 22, 27, 30–33, 38, 39, 41). The compiled estimates showed that the prevalence of shoulder injuries among delivery workers is 39% (95% CI: 0.27–0.52), reflecting substantial variability across countries, study populations, and working conditions Figure 3.

##### Neck

**Figure 4:**
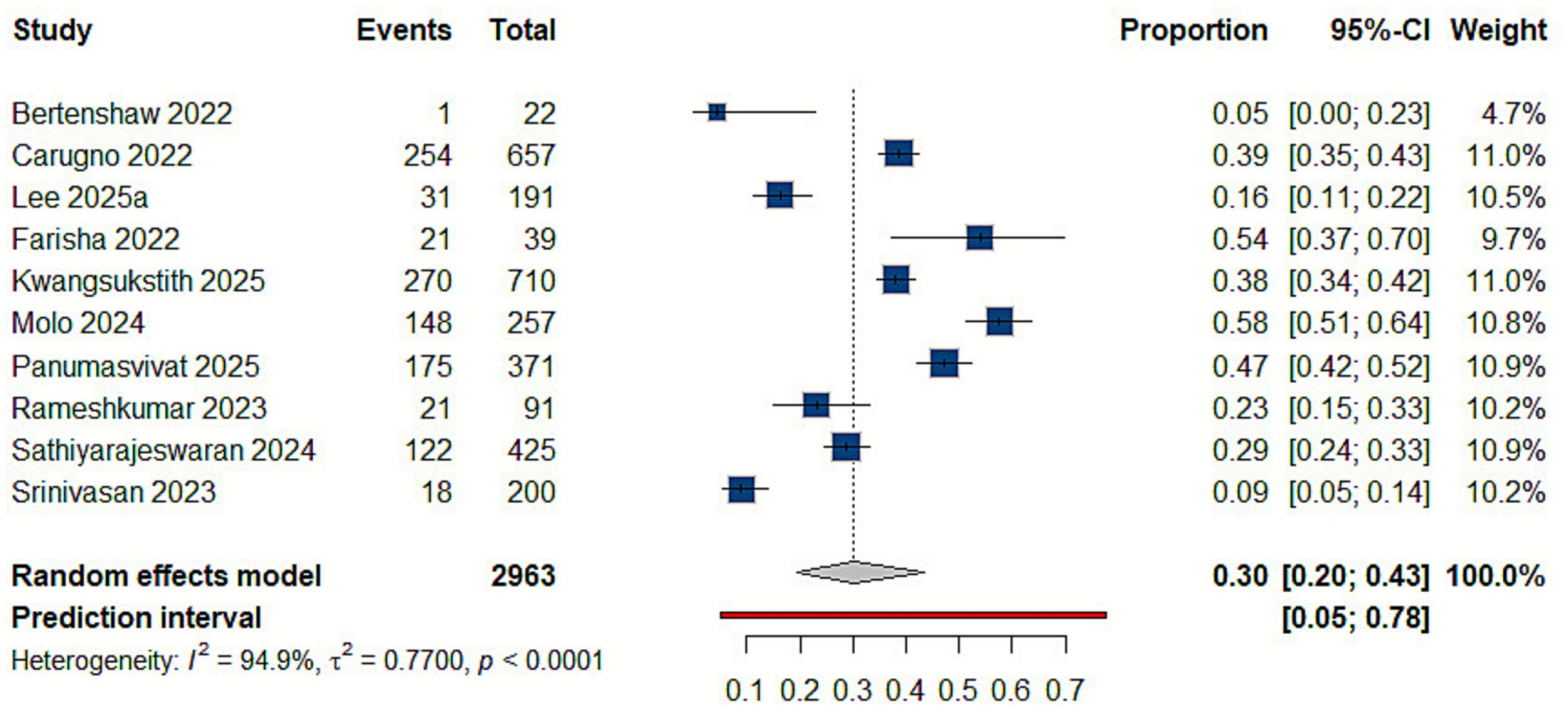
Forest plot of the prevalence of neck disorders.

Our analysis revealed that the prevalence of neck disorders among delivery workers, as demonstrated in ten studies (10, 21, 22, 27, 30–33, 38, 39), is 30% (95% CI: 0.20–0.43), reflecting substantial variability across countries, study populations, and working conditions. Figure 4.

##### Upper back

**Figure 5:**
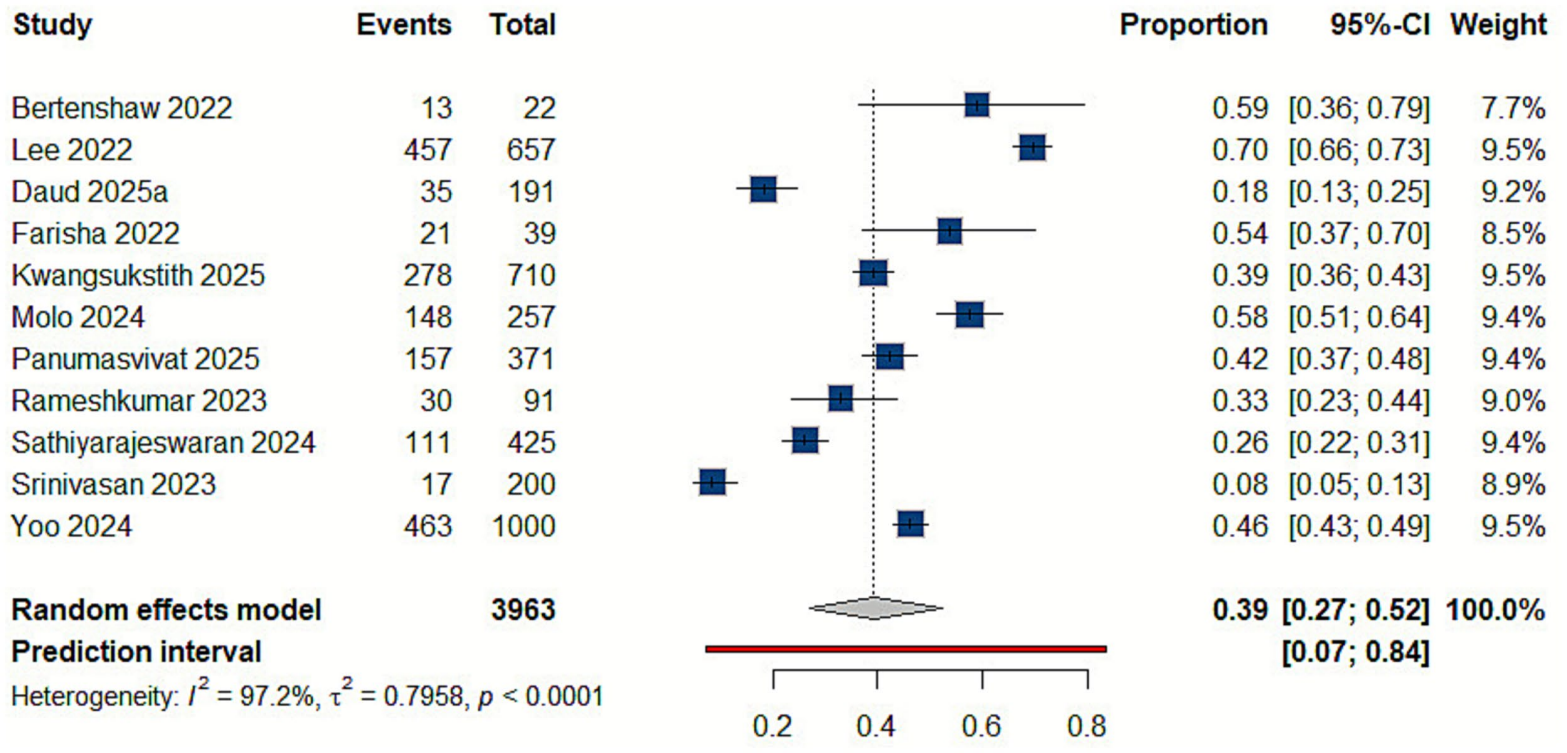
Forest plot of the prevalence of upper back disorders.

Combining data from nine sources on upper back disorders among delivery workers yielded a pooled prevalence of 24% (95% CI: 0.14–0.39), reflecting substantial variability across countries, study populations, and working conditions (10, 21, 22, 27, 30, 31, 33, 38, 39) Figure 5.

### Road traffic accidents (RTAs)

**Figure 6:**
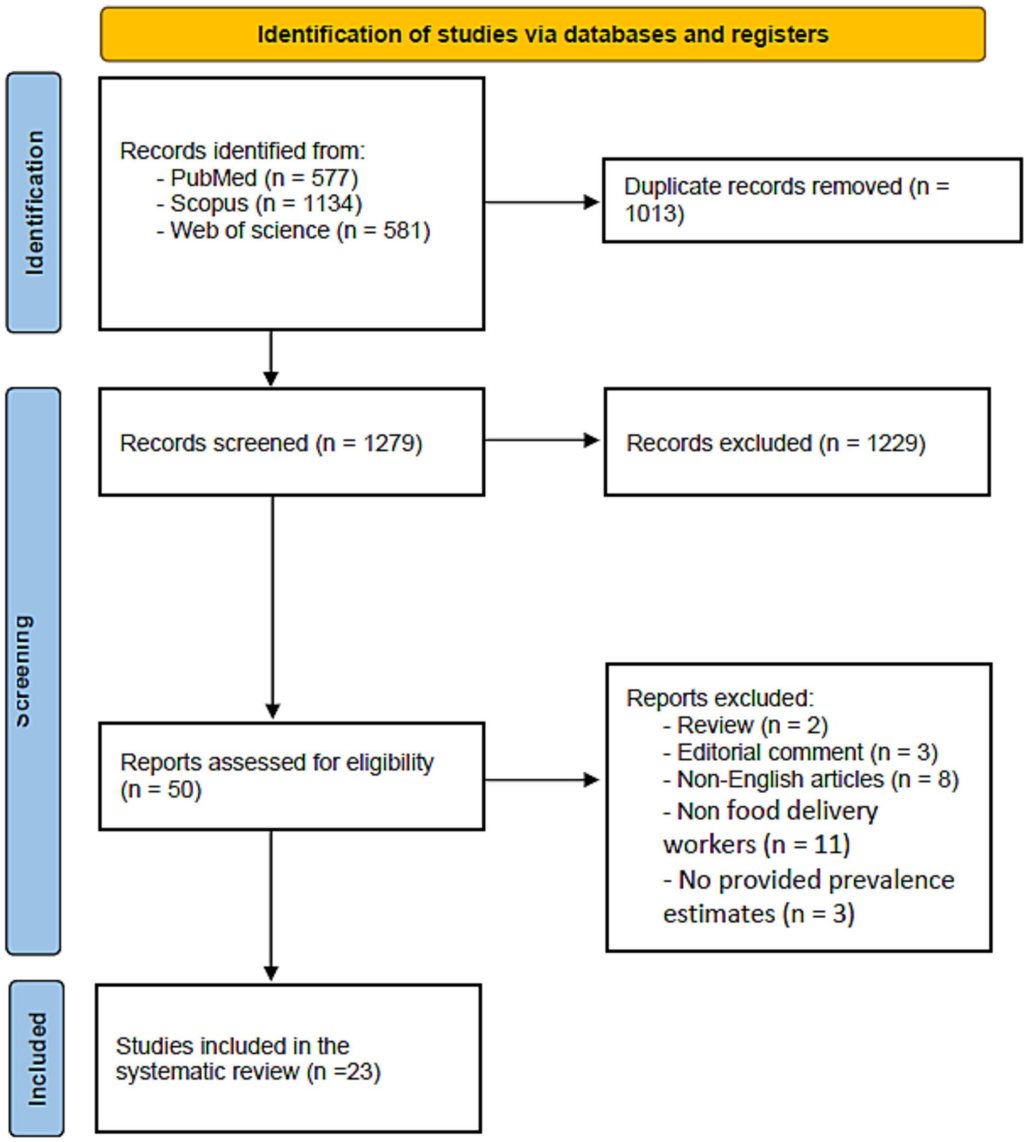
Forest plot of the prevalence of road traffic accidents (RTAs).

Data aggregated from 11 studies demonstrated a 25% prevalence rate of RTA (95% CI: 0.24–0.26) (9, 10, 21, 22, 28, 29, 31, 34, 36, 38–41) Figure 6.

#### Forms of violence against delivery workers

Violence-related outcomes reported in the included studies are summarized below.

##### Physical violence

Boniardi et al. reported that 12% of workers were exposed to physical assault, with 48% of those fearing for their health due to the physical encounter (9). Laskaris et al. reported that the overall prevalence of physical assault among delivery workers was 20.8%. For full-time workers, it was 25.3, and 11.7% for part-time workers. Moreover, they noted that 39.9% of those who were exposed to physical violence sustained significant injuries due to the encounter (29). Yoo et al. reported a lower rate of physical assault among food delivery workers (3.5%) (41). Physical violence was often due to direct physical attacks (9, 29), attempted vehicular theft (9, 29), and hit-and-runs (24).

##### Verbal violence

In a study by Boniardi et al., verbal assault was reported by 28% of the workers (9). Yoo et al. noted that food delivery workers were often exposed to verbal abuse (54%), threats (15%), and insults (55%) (41). These were often in the form of insults due to road rage, harassment, and mistreatment from employers and customers (9, 27, 36, 41).

##### Psychological violence

Among gig workers, substantial psychological distress is caused by time pressures, overworking, financial instability (9, 24, 29, 41), unfair customer ratings (41), exploitation, lack of worker protections, customer dissatisfaction, job stressors, and limited rest leading to anxiety, anger, fatigue, and depression (28, 32, 40, 41). Racial discrimination and language barriers are especially burdensome for workers of foreign, ethnic, or non-English speaking backgrounds (9, 29).

## Discussion

After synthesizing the current evidence, we noted that delivery workers face occupational hazards with a high prevalence of various work-related injuries. For instance, lower back injuries were the most prevalent (43%), followed by shoulder injuries (39%), neck (30%), and upper back (24%). Our analysis revealed a high prevalence of road traffic accidents (RTA; 25%).

All pooled prevalence estimates in this meta-analysis were characterized by very high heterogeneity (*I*^2^ values exceeding 95%), indicating substantial between-study variability. This heterogeneity likely reflects differences in geographical settings, traffic environments, vehicle types, working hours, recall periods, and measurement instruments across studies. As a result, the pooled prevalence values should be interpreted as indicative of the overall burden of WMSDs among food delivery workers, rather than as precise estimates applicable to any single context.

We assessed the records to ascribe risk factors for occupational injuries among delivery workers, which evidently operated at multiple levels. Gig economy or platform work itself, as an occupational model, is a fundamental driver of occupational hazards. Moreover, a system where the risk of injury is amplified is created by precarious employment, lack of worker protection (health insurance, accident tracking, safety education), algorithmic management enforcing intense time pressure, and financial incentives promoting dangerous practices, such as speeding, ignoring unsafe weather or road conditions. On the worker’s level, young males with low education and income were more likely to experience occupational accidents, whereas older workers were more likely to experience musculoskeletal disorders. Other negative behavioral aspects, such as irregular meals, dehydration, and poor sleep, all strongly contribute to work-related injuries, chronic pain, and musculoskeletal disorders. In addition, open-door vehicles (motorcycles, bicycles, e-bikes, etc.) all increase the risk of musculoskeletal disorders and traffic accidents among delivery workers.

Our findings closely align with the conceptual framework proposed by Taylor et al., who described gig work as a system characterized by work intensification, algorithmic management, economic precarity, and effort–reward imbalance. Within this system, workers are continuously incentivized to maximize productivity while absorbing occupational risks individually, with limited institutional protection. This model helps explain why food delivery workers in our review exhibit high prevalence of musculoskeletal disorders, traffic accidents, and psychological distress, as structural pressures encourage prolonged exposure to physical load, fatigue, unsafe traffic practices, and chronic stress. Importantly, Taylor et al. emphasized the normalization of risk in gig work, whereby hazardous conditions become perceived as unavoidable or acceptable, potentially contributing to underreporting of injuries and delayed healthcare seeking among delivery workers (17).

To contextualize our systematic review findings, evidence from related populations outside the present review suggests similar injury patterns among bicycle couriers and commercial cyclists. For example, Dennerlein et al. relayed data supportive of this, as they showed that bicycle couriers were at a higher risk of dislocations, sprains, musculoskeletal strains, and traffic accidents. Importantly, 70% of workers reported losing days of work, while 55% reported needing medical care for their injuries; however, only 24% of the sample were regularly using protective equipment. They inferred that 66% of the injuries sustained during riding were related to attempting to avoid crashes with closed vehicles or pedestrians (42). Heyer et al. specifically touched upon the higher incidence of injuries among delivery workers who use electric bikes, which can generate greater speed than regular bicycles (43).

Ergonomically, the two-wheeled vehicles used by delivery workers to boost speed and swift maneuvering in traffic are intrinsically dangerous for the same reasons, as the rider is exposed to greater collision risks, especially in riders who are not properly equipped with safety measures (e.g., helmets). Although these may allow workers to complete their tasks faster, they pose significant safety concerns, especially in the case of electrically modified traditional bikes, which do not abide by the safety regulations of original e-bikes (9).

According to Rusli et al., in the first half of 2017, Shanghai witnessed 76 fatal traffic accidents involving delivery workers, with one delivery rider dying every 2.5 days. This increased to more than 150 crashes by 2020. On a separate note, they highlighted that the need for better ratings and less customer dissatisfaction often incentivizes traffic violations committed by food delivery workers. Upon examining the data of 225 delivery riders, they noted that those who were involved in traffic accidents were often young, full-time workers, with a work experience of <2 years, compared to those who were not involved in crashes (*p* < 0.01 for all). Additionally, traveling a daily distance of 100 kilometers or more was significantly associated with higher odds of being involved in traffic crashes (OR 1.79, 95% CI: 1.04–3.10, *p* = 0.04) (44). Shin et al. observed a significant association between traffic violations among injured delivery riders (who used motorcycles as their courier vehicle) and crashing with a vehicle of any type (*p* < 0.001) and crashing with a motorcycle (*p* = 0.018) (45). Byun et al. stated that novice delivery riders (who use motorcycles) often do not get enough training before starting the job, leading to significant traffic violations and injuries (46).

Mateos-Gonzalez et al. (47) hypothesized a direct relationship between job insecurity and the development of musculoskeletal disorders. In their correlation model, job insecurity (measured by the job insecurity scale) showed significant correlation with psychosocial distress (as measured by the job content questionnaire). Although this correlation was weak, it reached statistical significance (*r* = 0.108, *p* < 0.05). Moreover, they found a direct link between work-related musculoskeletal disorders (WMSDs; as evaluated by the Nordic Musculoskeletal Questionnaire) and higher physical workload (*r* = 0.214, *p* < 0.001), as well as job insecurity (*r* = 0.136, *p* < 0.05). This suggests that occupations with no guarantee of workers’ financial safety may influence the development of musculoskeletal disorders, with the physical labor of the job being a mediating factor (47). Taylor et al. emphasized the impact of job security on gig workers in a prior systematic review, revealing that it led to feelings of uncertainty among workers, often pushing them to overwork and compromise their health, and allowing them to fall into exploitative practices by their employers. Moreover, they found a significant negative correlation between worker health and the pay structure of the gig economy, verifying the deleterious impact of insecure payment structures (17).

Other than accidental injuries, delivery workers also face customer and employer abuse in the forms of physical (direct attacks, theft, and hit-and-runs), verbal, and psychological violence. Reported prevalence of physical assault among delivery workers varies (3.5–25.3%), likely reflecting different regional contexts, but the consequences are severe, with nearly 40% of assaulted workers sustaining significant injuries and many fearing for their health. Similarly, high rates of verbal abuse (54%), insults (55%), and threats (15%) are reported, stemming from road rage, customer dissatisfaction, and employer mistreatment. Such intense work conditions lead to psychological distress among workers, especially with unfair customer ratings, discrimination, and time pressures, all leading to depression and anxiety. The over-representation of vulnerable populations (immigrants, minorities, low-income individuals) in this workforce suggests these hazards disproportionately affect marginalized groups. In a study by Mbare et al., evaluating a group of 20 immigrant delivery workers, they criticized the badge systems of platform jobs as they instate unfair criteria for high worker rankings. For instance, the evaluation criteria for delivery workers included working on weekends, task completion speed, and tardiness. Tardiness was evaluated based on logging into the platform application from a location other than the one where the gig is assigned. Two workers reported that their platform removes their ranking if they reported they were sick and could not do a certain job, which later lowers their chances of obtaining more shifts, causing considerable feelings of job insecurity and leading them toward unsafe practices, such as working while sick (48).

As van Doorn et al. stated, the ambiguity of the gig economy poses grave concerns regarding worker protections, as those platforms often take advantage of the legal loopholes of platform labor to pay workers below the regional minimum wage with no guarantee that one will remain hired (49). Moreover, Ticona and Mateescu indicated that despite the effort to formalize platform work, this remains only a façade, as these platforms do not impose formal employment regulations, altering the culturally recognized concept of employment. They argued that this casualization of employment could be hazardous to workers’ wellbeing, especially with the use of rating systems that are strategically constructed to evoke a reaction from consumers and define the categories by which workers are evaluated, which is often unjust (50).

To our knowledge, this is the first systematic review and meta-analysis to quantitatively estimate pooled prevalence rates of work-related musculoskeletal disorders across specific anatomical regions among food delivery workers, while also providing a narrative synthesis of road traffic accidents and workplace violence affecting this workforce. We also detailed the impact of the gig economy model on the physical and psychological wellbeing of delivery workers. The findings of our work are impacted by limitations inherent to the included studies, such as the cross-sectional design, potential recall and reporting bias due to the reliance on self-reported data, especially for sensitive issues like violence, as well as potential underreporting by precarious workers. In addition, heterogeneity in measurement tools and definitions across studies and geographic concentration limited global generalizability. Variability in recall windows and assessment instruments across studies likely contributed to methodological heterogeneity and may have influenced the pooled prevalence estimates. This heterogeneity should be considered when interpreting the summary results. Moreover, the exclusion of non-English studies and abstract-only publications (i.e., records not available in full text) may have introduced language and publication bias, as conference abstracts often lack sufficient methodological detail and outcome reporting to allow inclusion in quantitative synthesis. This may have resulted in underrepresentation of some relevant studies, particularly from non English-speaking regions. Also, our search strategy may have been limited by the use of specific occupational labels (e.g., “food delivery workers”), which could have excluded relevant studies using alternative terms such as “courier,” “rider,” or “driver” without explicit reference to food delivery. This restriction may have led to under-identification of some eligible studies and should be considered when interpreting the findings. However, we attempted to mitigate this risk through extensive manual screening, including forward and backward reference checking and repeated manual review of related literature.

## Conclusion

In conclusion, our study emphasized the significant occupational hazards faced by delivery workers, including high rates of musculoskeletal injuries, traffic accidents, and workplace violence. These risks stem from systemic factors such as precarious employment, time pressures, inadequate safety measures, and exploitative gig economy structures, compounded by individual, external, and behavioral factors. Urgent interventions, including stronger labor protections, ergonomic improvements of vehicles, safety training, and fairer platform policies, are needed to mitigate these risks and safeguard workers’ wellbeing. Addressing these challenges requires multi-level reforms to ensure safer and more equitable working conditions in the gig economy.

## Data Availability

Publicly available datasets were analyzed in this study. This data can be found at: all data generated or analysed during this study are included in the published article.
