# Supplementary material for: Work-related musculoskeletal disorders among gig-based food delivery workers: a systematic review and meta-analysis
Source: Front Public Health. 2026 Mar 23;14:1788523. doi: 10.3389/fpubh.2026.1788523 (PMC13050918; doi:10.3389/fpubh.2026.1788523)
Supplement: Supplementary file 1 [file supplementary_file_1.docx]

**Supplementary Appendix 1**: Detailed search strategy for the retrieved databases.

| **Appendix A**: databases were used to search for articles related to the following key words: | | |
| --- | --- | --- |
| **Databases** | **Search Strategy** | **Results** |
| **Pubmed** | (("food" OR "gig") AND (worker OR workers OR deliverer OR delivery OR "takeaway riders"))  AND ("musculoskeletal disorders" OR MSDs OR WRMSDs OR WMSDs OR pain OR injuries OR discomfort) | 577 |
| **Scopus** | (TITLE-ABS-KEY(("food" OR "gig") AND (worker OR workers OR deliverer OR delivery OR "takeaway riders")))  AND (TITLE-ABS-KEY("musculoskeletal disorders" OR MSDs OR WRMSDs OR WMSDs OR pain OR injuries OR discomfort)) | 1134 |
| **Web of science** | TS=(("food" OR "gig") AND (worker OR workers OR deliverer OR delivery OR "takeaway riders"))  AND TS=("musculoskeletal disorders" OR MSDs OR WRMSDs OR WMSDs OR pain OR injuries OR discomfort) | 581 |
